# Deep Green Diagnostics: Urban Green Space Analysis Using Deep Learning and Drone Images

**DOI:** 10.3390/s19235287

**Published:** 2019-11-30

**Authors:** Marco A. Moreno-Armendáriz, Hiram Calvo, Carlos A. Duchanoy, Anayantzin P. López-Juárez, Israel A. Vargas-Monroy, Miguel Santiago Suarez-Castañon

**Affiliations:** 1 Instituto Politécnico Nacional, Centro de Investigación en Computación, Av. Juan de Dios Bátiz s/n, Ciudad de México 07738, Mexico; hcalvo@cic.ipn.mx (H.C.); duchduchanoy@gmail.com (C.A.D.); 2 Cátedra CONACyT, Instituto Politécnico Nacional, Centro de Investigación en Computación, Av. Juan de Dios Bátiz s/n, Ciudad de México 07738, Mexico; 3 Escuela Superior de Cómputo, Instituto Politécnico Nacional, Av. Juan de Dios Bátiz s/n, Col. Lindavista, Ciudad de México 07738, Mexico; paop9503@gmail.com (A.P.L.-J.); vargasmonroyisraelagustin@gmail.com (I.A.V.-M.); sasuarez@prodigy.net.mx (M.S.S.-C.)

**Keywords:** deep learning (for social good), remote sensing, biomass analysis

## Abstract

Nowadays, more than half of the world’s population lives in urban areas, and this number continues increasing. Consequently, there are more and more scientific publications that analyze health problems of people associated with living in these highly urbanized locations. In particular, some of the recent work has focused on relating people’s health to the quality and quantity of urban green areas. In this context, and considering the huge amount of land area in large cities that must be supervised, our work seeks to develop a deep learning-based solution capable of determining the level of health of the land and to assess whether it is contaminated. The main purpose is to provide health institutions with software capable of creating updated maps that indicate where these phenomena are presented, as this information could be very useful to guide public health goals in large cities. Our software is released as open source code, and the data used for the experiments presented in this paper are also freely available.

## 1. Introduction

Grass is perhaps the most extended plant on earth. Except for rainforests, we may find grass everywhere on the planet [1]; it grows even in saltwater and arid areas. Grass plays a fundamental role in our ecosystem and has a significant impact on agriculture and economy [2]. For instance, a very substantial percentage of meat production comes from ruminant animals that are fed with grass [1]; the grass system roots help to keep the soil stable and prevent erosion [2]. For those of us who live in urban zones, grass is fundamental to our well-being [3,4]. Some studies have shown that natural landscape has a positive influence on mood, stress levels, cognitive function, and impulse control [5]. Furthermore, having access to green areas can help to some extent to prevent serious health problems such as obesity, heart disease, and diabetes, because these environments are more attractive for people to exercise and do outdoor activities [4].

Most studies of urban green space used the Normalized Difference Vegetation Index (NDVI) derived from satellite imagery [6]. For instance, in [7] the authors found, using data from the U.S.-based Nurses’ Health Study prospective cohort and the NDVI, that exposure to greenness is associated with decreased mortality and improves health. In [8], the NDVI was used in Barcelona, Spain, to conclude that exposure to outdoor surrounding greenness was associated with a beneficial impact on cognitive development in schoolchildren in the second to fourth grades (7–10 y.o.). In [9], the authors used both a cohort study, consisting of approximately 1.3 million adults, and the Cox proportional hazards models in order to, respectively, assign estimates of exposure to greenness derived from remotely sensed NDVI (NASA’s Aqua satellite), and estimate associations between residential greenness and mortality. They concluded that increased amounts of residential greenness were associated with reduced risks of dying from several common causes of death among urban Canadians. Finally, we mention that in [10], using different tools, the authors also found evidence that supports that, among other factors like food quality and exercise, being exposed to greenness improves health— particularly, the risk of heart disease can be reduced. Although the NDVI is useful for quantifying vegetation, because of its resolution, it is not able to detect smaller details, such as the presence of cans, plastic bags, bottles, etc., so is not suitable for our purposes.

Identifying contamination is important because contaminated locations may represent places with health risks and possible proliferation of harmful fauna. Automatic detection of contaminated land in the city can be very useful information to design forecast models for solid waste generation, as in [11], where the authors use four different Artificial Intelligence techniques to model this serious problem. In [12], the pollution problem is considered part of the Sustainable Development Goals for Smart Cities (SC). These cities seek to promote economic development, improve the welfare of their citizens, and help in the ability of people to use technologies to build sustainable services.

## 2. Related Work

**Figure 1:**
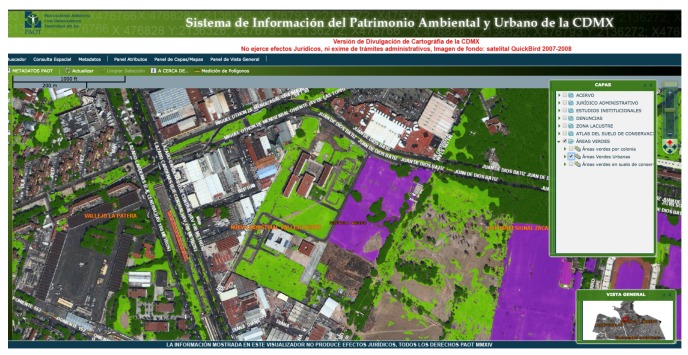
*Sistema de Información del Patrimonio Ambiental y Urbano de la Ciudad de México* (SIPAU-CDMX) system interface [15].

Regarding previous literature dealing with tasks similar to the main issue of this study, we found some works that use México City’s images. In [13], the authors use images from the Landsat and RapidEye satellites, with resolutions of 30 m per pixel and 5 m per pixel, respectively, to carry out a rural and urban grounds sustainable graphic analysis. To this end, the authors establish six classes for ground classification (urban ground, forest, water, plain ground, grass, and clouds) using the eCognition commercial software [14]. As a conclusion, they mention that low-resolution images are not adequate for ground’s identification in some regions of the city. Another one is the System of Information on Environmental and Urban Assets (*Sistema de Información del Patrimonio Ambiental y Urbano de la Ciudad de México*—SIPAU-CDMX) [15]. This system classifies terrain among three different types of green areas: wooded areas, grasses or shrubs, and sports fields, using images from the QuickBird satellite (2007–2008) as background images, see Figure 1. However, the system does not provide any information about vegetation health or the existence of some pollution on the ground.

Due to the relatively low resolution of the satellite images for our purposes, we decided to experiment with the use of an unmanned aerial vehicle (UAV). A brief description of some related works using this kind of vehicles is presented below.

In [16], the authors introduce an automatic content-based analysis to detect and mark objects arbitrarily placed on the ground from high-resolution aerial images. Their analysis relies on a novel two-stage-training convolution neural network. In a different work, because of its feasibility and availability, a UAV low-height remote sensing technique has been used [17]. They propose an extraction method for cultivated land information based on deep convolutional neuronal network and transfer learning. The method was used for identifying cultivated land with an experimental accuracy of 91.7%. On a different application, fruit detection is a significant issue in food production. The convolutional neural network (CNN)-based techniques to solve this matter are deeply improved when the engine is fed with aerial images taken using UAV. In [18], the authors developed a methodology to assess the health of a citrus plant. To this end, a combination of images taken from a UAV, and data collected from sensors in the ground, are used to train four models, CNN included.

Even when lawns provide several benefits to people and the environment, some risks are inherent, the most evident being water consumption. Determining the quality of grass is critical to avoid the overuse or underuse of water. Focused on this problem, the authors in [19] develop an approach to find out the quality and color of turf grass to assist in the optimization of water consumption. Their main algorithm is Deep Learning (DL) based, together with images taken from UAVs. Another interesting turf grass quality identification methodology was developed in [20]. The images used in such work, taken with an RGB camera mounted on a UAV, were fed into a neural network (NN) to extract intensity, color, and texture, to eventually assess the turf grass quality according to its greenness. Finally, we mention two interesting works aimed to identify two types of vegetation landscapes. In [21], the authors propose a method that combines Random Forest and texture analysis for vegetation mapping in urban landscapes, based on images captured using a UAV. The method classifies lawn, trees, and shrubs. Another study is [22], where the authors’ main goal is to identify undesirable weeds based on convolutional DL; they developed a detection software, which exploits the Simple Linear Iterative Clustering (SLIC) superpixel algorithm to segment plantation images captured by UAV’s.

As far as we know, the problem of verifying simultaneously the land’s health along with the presence of contamination has not been specifically addressed. This is why we present a novel methodology for urban green spaces classification using a two-level system: first we consider the level of health of the land, and then the presence of contamination. In this way, two problems of different nature and level of severity that commonly occur together in the landscape are tackled using aerial images of the terrain taken by a UAV. This latter goal serves the purpose of saving costs of acquiring high resolution satellite imaginery—which, as we have discussed, may not be accurate enough in time and detail—and complements our goal of enabling people and institutions with limited resources to benefit from our solution, as well as to customize it, by releasing the implementation of our system as open source.

We organize the rest of this work as follows. In Section 1 we establish the problem to be solved. In Section 2 we reviewed what we consider the most relevant related work with our proposal. In Section 3 we explain in detail the architecture of our system, the design of the Deep Neural Network (DNN), and how the system presents the classification results. In Section 4 we give details about the model’s performance. Finally, Section 5 is devoted to the concluding remarks.

## 3. Deep Green Diagnostics (DGD)

**Figure 2:**
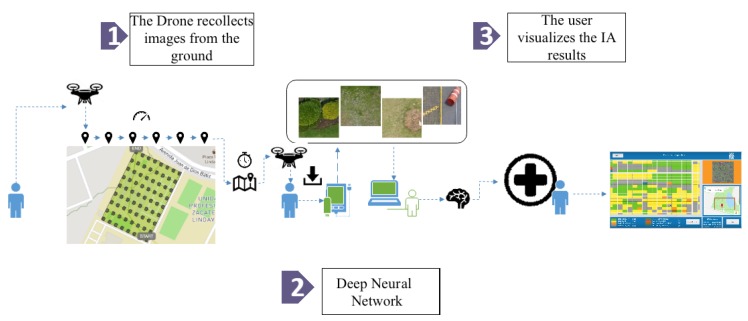
Deep Green Diagnostics general architecture.

**Figure 3:**
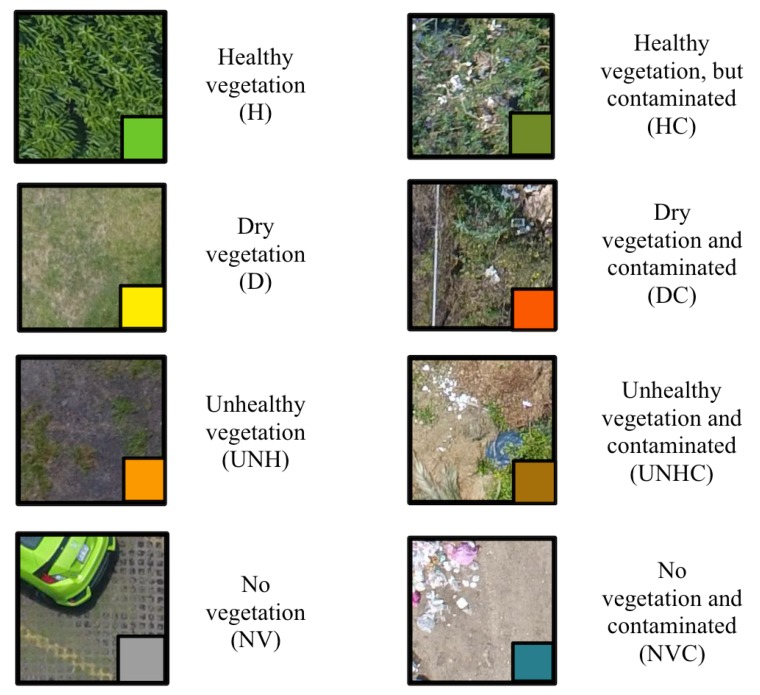
The eight proposed classes of terrain.

Our proposal consists of the modules shown in Figure 2, explained in detail below:

1. **Geotagged image dataset**. Users must fly a UAV about 30 m above ground level to obtain the image of the terrain to be analyzed; these images include the georeference in their metadata and will constitute the dataset used for training the Deep Neural Network. See Section 3.1 for more details.
2. **Deep Neural Network**. Users enter the previously obtained dataset into the system, which in turn will process it using the architecture described in Section 3.2. As output, the system will classify each image according to the eight classes shown in Figure 3.
3. **Visualization and retraining**. In this stage, the users are able to visualize the classification results and, if desired, re-train the pre-loaded DNN with a new dataset. Details are presented in Section 3.3.

### 3.1. Geotagged Image Dataset

The process starts by capturing a set of aerial images, recently taken from ground-level, within a distance of 20 to 30 m and with natural light, ideally on a sunny day between 7 a.m. and 5 p.m. The images must be georeferenced and saved into the RGB format. For this purpose, we use a UAV, the DJI Phantom 4 drone [23], to obtain a dataset that meets the required characteristics.

**Figure 4:**
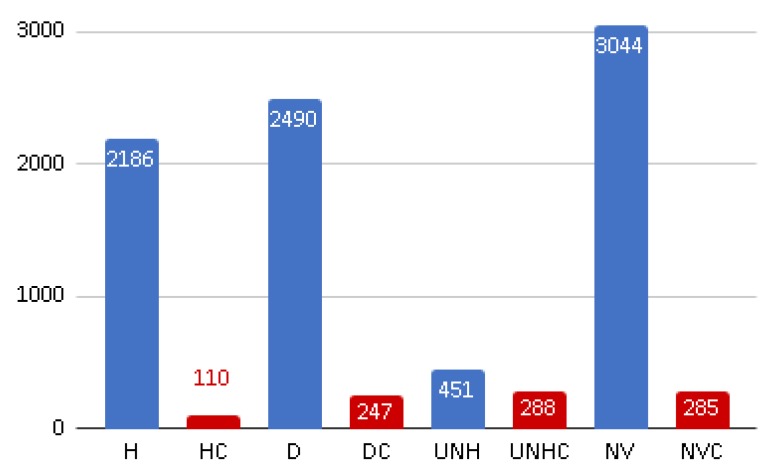
Number of data in each of the classes for training.

After several flights of the following landscapes, a park (Bicentenario Park), a university campus (ESCOM-IPN), suburban neighborhood (Bonito Ecatepec), and a forested area were selected for designing our solution. The obtained images (img_drone) are divided into a regular grid to obtain images of 200 × 200 pixels each (img_200 × 200), thus obtaining more than 135,000 images of this size. In order to carry out the labeling, 9901 images were randomly taken. Labeling consists of assigning a tag to indicate the state of health of the soil; if the soil is also contaminated, another tag is added. The ground health classes are: healthy vegetation (H), dry vegetation (D), unhealthy vegetation (UNH), and no vegetation (NV). The latter refers to any other type of objects, such as pavement, buildings, cars, among others. Then, contaminated images are marked, adding the letter C to the previous classes: HC, DC, UNHC, and NVC, which, along with the ground health condition, indicate the presence of garbage contamination, obtaining eight classes of terrain. Examples of these classes are shown in Figure 3 and the statistics in Figure 4. This dataset is available in [24].

### 3.2. Deep Neural Network

Before the software can be used, it is necessary to design a DNN capable of properly classifying an image of the terrain in one of the eight classes defined in Figure 3.

#### Model Training and Validation

**Figure 5:**
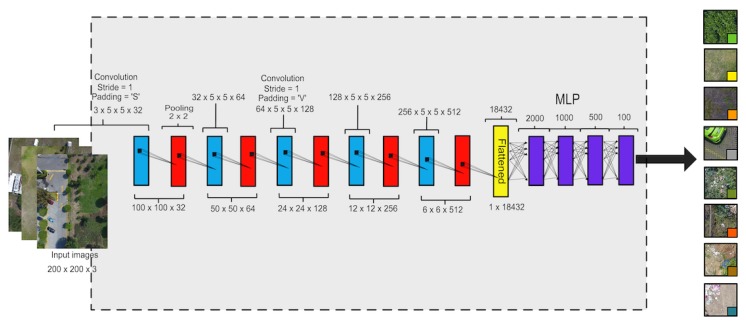
Architecture of the designed Deep Neural Network (convolutional neural network [CNN]+Multilayer Perceptron [MLP]).

To this end, we split our dataset into 9001 images for training and 901 for testing. Then, we used the grid search method to obtain a suitable DNN for our goal, and this architecture is shown in Figure 5. It is composed of a convolutional neural network (CNN) [25] which automatically extracts features from the land images and a Multilayer Perceptron (MLP) as a classifier.

The final parameters [26] used to train this DNN are shown in Table 1. During the experiments, we noticed that regularization does not help in the learning process, so we decided not to use it.

To evaluate the DNN’s behavior, we used the well-known and widely used metrics to evaluate the performance of this kind of networks—precision, recall, and F-score [27]—which are calculated in this context using the following equations. tp means true positives, tn true negatives, fp false positives, and fn false negatives.

(1) $$\begin{array}{ccc} Accuracy & = & \frac{\sum_{i=1}^{l}\frac{tp_{i}+tn_{i}}{tp_{i}+fn_{i}+fp_{i}+tn_{i}}}{l} \end{array}$$

(2) $$\begin{array}{ccc} Precision & = & \frac{\sum_{i=1}^{l}tp_{i}}{\sum_{i=1}^{l}tp_{i}+fp_{i}} \end{array}$$

(3) $$\begin{array}{ccc} Recall & = & \frac{\sum_{i=1}^{l}tp_{i}}{\sum_{i=1}^{l}tp_{i}+fn_{i}} \end{array}$$

(4) $$\begin{array}{ccc} F_{1}\text{\_}score & = & 2\times \frac{\left(Precision\times Recall\right)}{Precision+Recall} \end{array}$$

**Figure 6:**
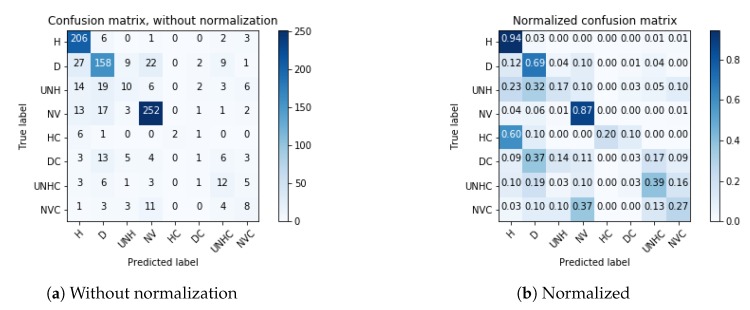
Confusion matrices for the test dataset.

Using the confusion matrix values shown in Figure 6a with l=8, we obtained the following results: training accuracy: 100%, testing accuracy: 72%, precision (micro avg.): 72%, recall (micro avg.): 72% and F${}_{1}$ score (micro avg.): 0.71%. Additionally, we show in Figure 6b the normalized confusion matrix, which allows to examine in detail the classifier’s behavior. In this figure, it can be seen that the Healthy (H) and the No-Vegetation (NV) classes obtained the scores 0.94 and 0.87, respectively.

It is important to notice that, since we acquire images at an altitude of 30 m, resolution is 1.31 cm/px. Images taken at low altitude have the advantage of having high quality, and allowing to easily spot small details, like garbage—pet bottles, organic waste, plastic bags, etc.

From each of the four selected terrains, we report the number of labels of each class for these fields (see Table 2). In Table 3 we show the ground surface covered by the drone, measured in square meters.

Finally, our open source software is available at [28], and a tutorial video about how to use it can be found at [29].

### 3.3. Visualization and Retraining

**Figure 7:**
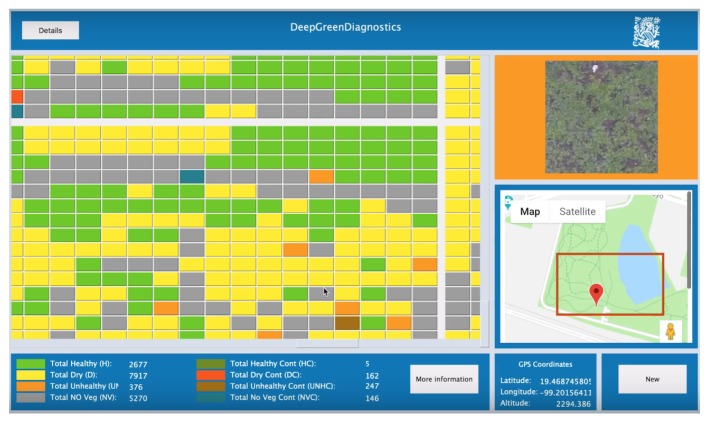
Visualization of results as matrix cells.

**Figure 8:**
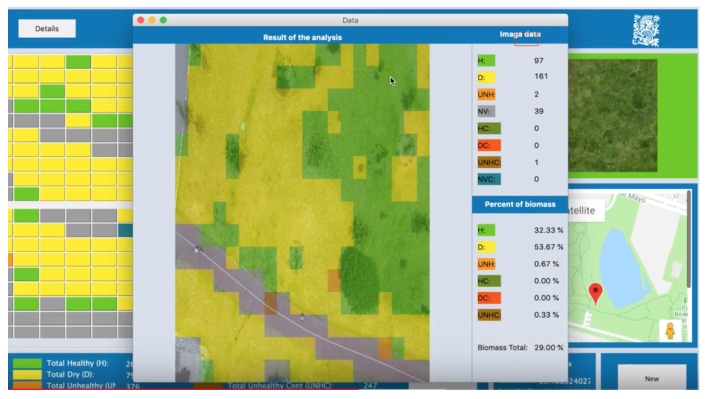
Visualization of detail and biomass percentages.

The output of the system is represented as cells arranged in a matrix. Each cell is colored according to the color code in Table Figure 3, as shown in Figure 7. Cells correspond to a portion of the analyzed terrain. To be able to see in detail the results of classification, it is possible to click on any cell of interest, then the system informs about the percentages of healthy terrain, percentage of biomass, etc. See Figure 8.

Also, there is a button labelled as retrain. This button allows the user to load a new dataset from a different terrain, and retrain the DNN. When the training is finished, the user can load data from this new terrain and obtain new classification results.

**Figure 9:**
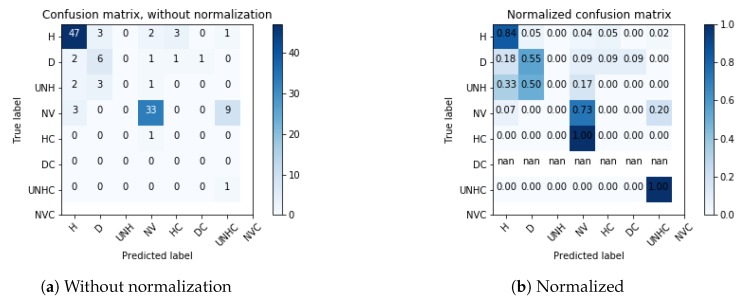
Confusion matrices for the test dataset of Figure A1.

**Figure A1:**
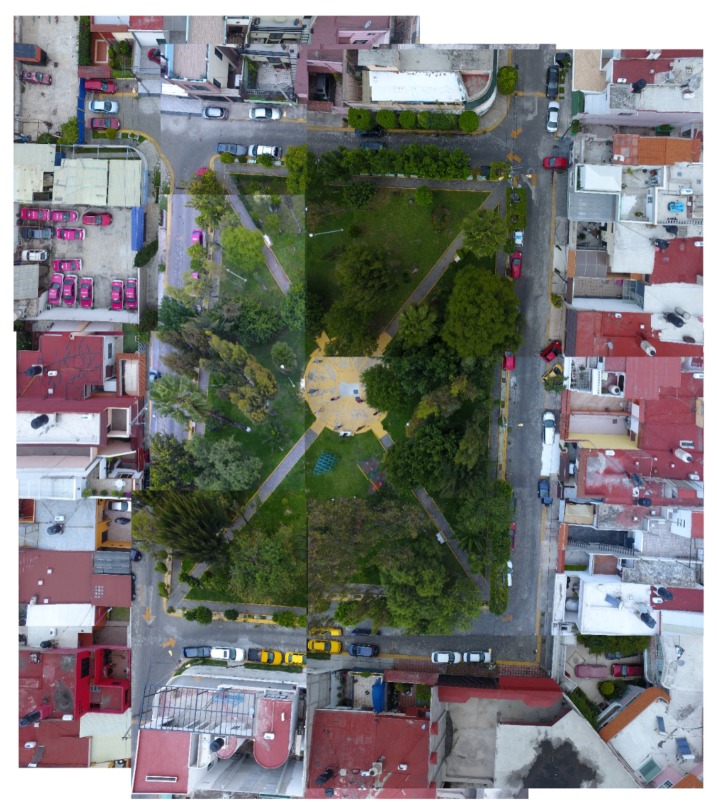
Map used for retrain the DNN.

As an example, consider the map shown in Figure A1. For this map we applied the process described in Section 3. We obtained 600 images (img_200× 200) for retraining and 120 images (img_200 × 200) for testing. We ran the experiment 10 times and an average accuracy of 68.33% was obtained on the test set. Additionally, Figure 9b shows that the Healthy (H) and No-Vegetation (NV) classes obtained scores of 0.84 and 0.73, respectively, which is consistent with the results previously shown in Figure 6b. This is an example of how our method is able to reasonably analyze new fields without the need to change its architecture or having to manually adjust further parameters. This is a convenient feature because this way it is possible for the final user to easily apply this method to obtain information from their own maps.

### 3.4. Full Map Display

In addition, our software allows users to examine in detail each piece of classification results on a map. In order to illustrate this, we manually built a complete map of the four locations to show that is possible to precisely identify zones where the terrain is dry, unhealthy, and/or contaminated, see Figure A3, Figure A4 and Figure A5.

## 4. Discussion

### 4.1. Model’s Performance

**Figure A2:**
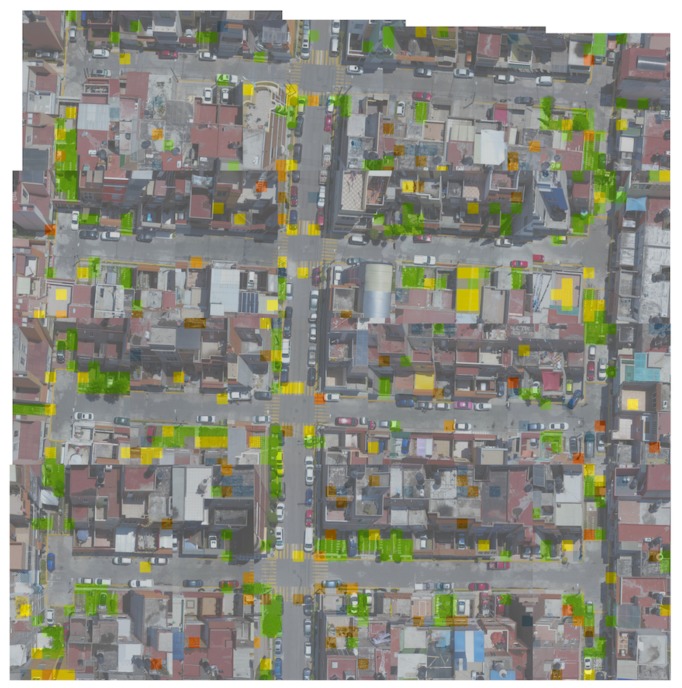
Classification results for Bonito Ecatepec.

**Figure A3:**
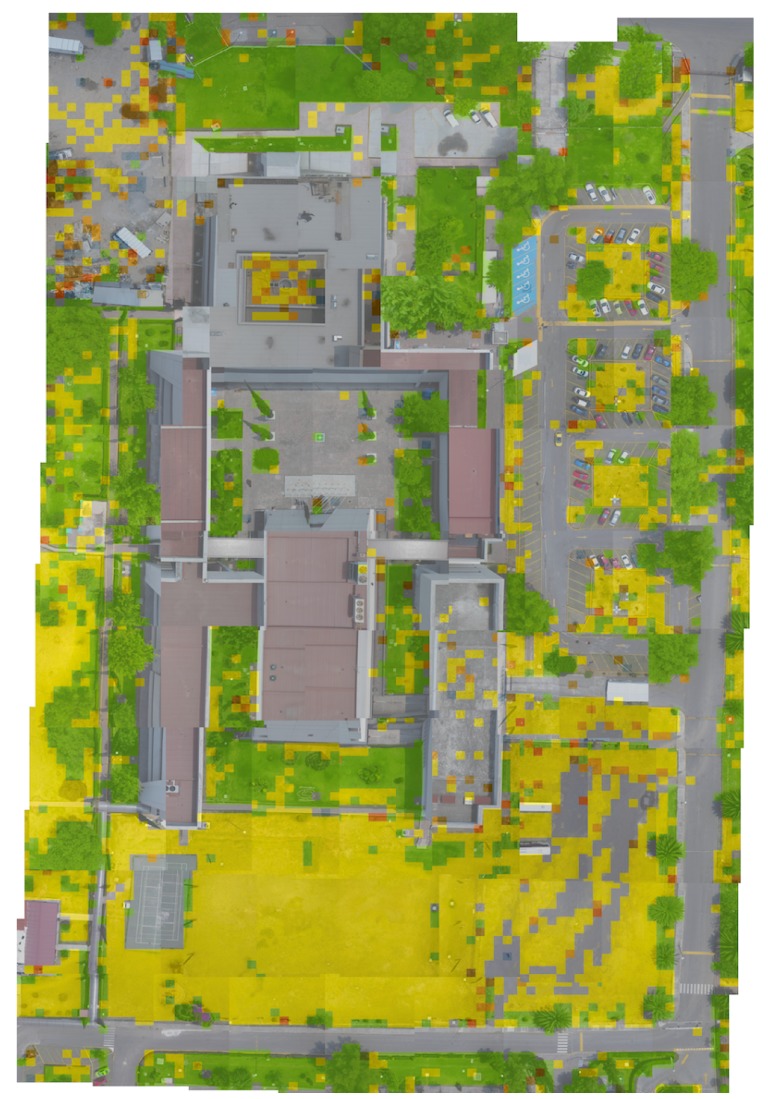
Classification results for ESCOM-IPN.

**Figure A4:**
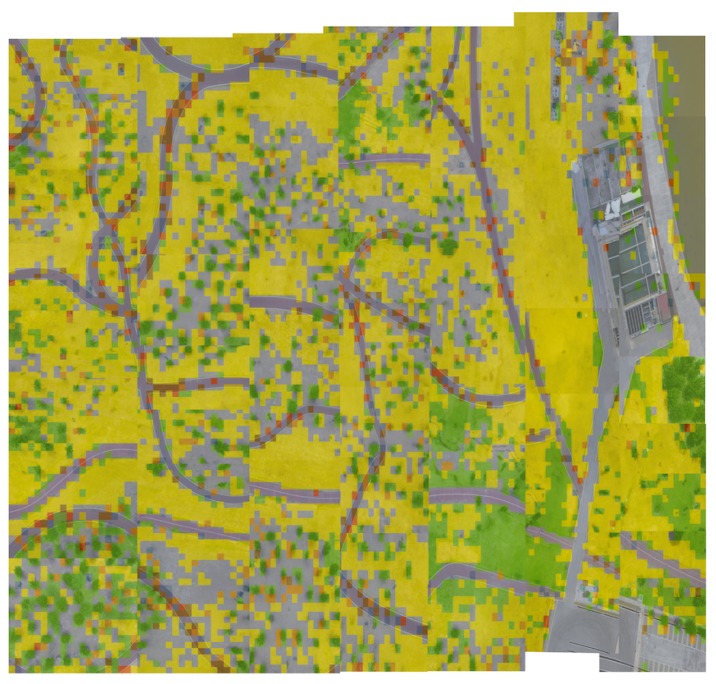
Classification results for Bicentenario Park.

**Figure A5:**
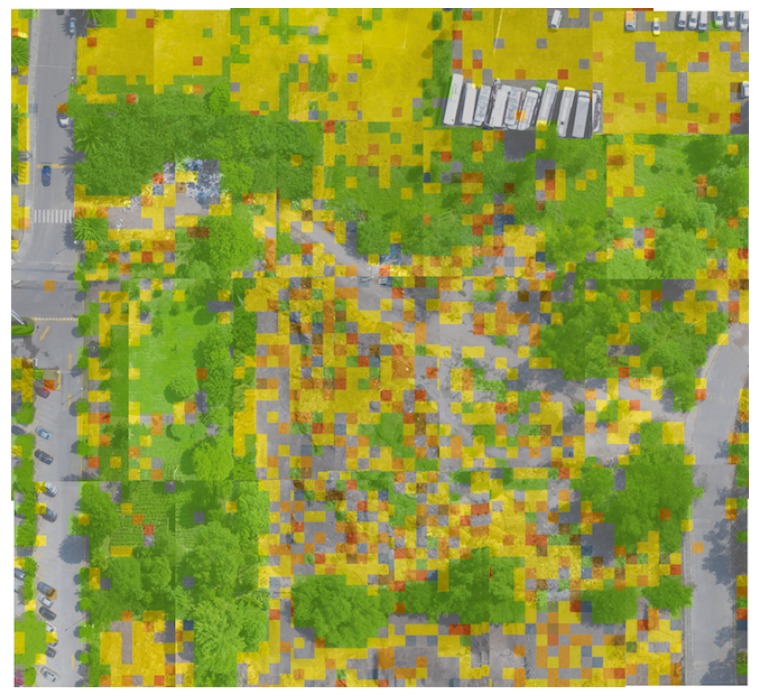
Classification results for a forested area.

In this study, the terrain classifier model obtains a testing accuracy of 72%. To our knowledge, there are no other works that perform exactly the same tasks, therefore, we are unable to present a straightforward comparison; however, there are several studies that use closely related approaches. For instance, in [30] the authors use the pre-trained network *Inception-v3* to extract the characteristics used to obtain land cover types, while in [31] a DCNN is used to extract from images a characteristic vector, to subsequently introduce it into an SVM classifier to generate the human perception mapping. To improve the knowledge capability of our system, we considered four terrains with very different or diverse landscapes, as shown in Figure A2, Figure A3, Figure A4 and Figure A5. From these terrains, we obtained a total of 49,800 image ( img_200 × 200) labels. The drone flights covered 341,822.18 m${}^{2}$. The obtained results support the evidence that our model can be used for knowledge transfer [32,33], i.e., it can be re-trained to add functionality into our system.

Additionally, in Table 4, we show the biomass percentage found in each terrain, and the percentage of the polluted area in all four reported maps, where we can see that the forested area has the greatest amount of biomass (78.69%), while Bonito Ecatepec is the most polluted (16.73%) and has the least amount of biomass (9.37%).

Terrain classification through modern technology has been carried out for several decades, because there are countless situations where the obtained information helps to solve or to avoid several problems. Initially, satellite images were used, whereas recently cameras mounted on unmanned aerial or ground vehicles are used as the primary source of images. Even when a full review of this topic is beyond the scope of this work, we mention in Table 5 some interesting studies related to terrain classification, with different image sources and applications, to emphasize its relevance. In [34], the authors analyze the health of tropical forests and one of their most important results is that the use of NDVI was less precise due to the saturation effect. Mainly due to this effect, we did not consider NDVI to be useful for our application.

Even when satellite information has been a reliable source of vegetation quantification, it is not an affordable source for most people. Conversely, images obtained from drones are of relatively easy acquisition, and they have the requisite quality to be used in several classification processes.

### 4.2. Software

Our software does not require measurements or adjustments of any kind, requiring only the images obtained by the drone flying over the terrain of interest to carry out the corresponding analysis. As far as we know, there is no other software that performs the same task, the closest software being SIPAU-CDMX [15], which we introduced in Section 1, identifying its differences to our system. Another similar software is called Atfarm [43], which, using satellite images, is able to analyze terrain using the Adapt-N algorithm. This algorithm indicates which parts of the farmland need to be fertilized. This software is exclusively oriented to agriculture precision, and consequently, it is not able to find pollution in the terrain, if it exists.

## 5. Conclusions

In this work we have shown that, using images taken by a drone at an altitude of around 30 m, it is possible to carry out detailed health and pollution analysis of a terrain. The successful design of the deep learning architecture allowed the system to perform a proper classification of previously unseen terrain images.

As we are using a well-established architecture, our main focus is on the proposal of a practical approach to apply deep learning in order to carry out environmental impact studies with high resolution (1.31 cm/px). Our main contribution consists of showing how using an unmanned aerial vehicle and deep learning can aid to perform automatic terrain health and contamination level analysis. We have introduced open source software useful to researchers or government agencies that require this kind of analysis. Related to future research, new images from other terrains could be obtained, particularly for highly polluted areas. Having more samples of each of the four classes that show polluted terrain parts may help to improve the DNN’s performance presented in this work in general. In summary, our results are trustworthy, but it is still necessary to expand the research and undertake analysis with more images of contaminated terrains.

## Figures and Tables

**Table 1:** Parameters used to train the Deep Neural Network (DNN).

General Parameters	Values
Batch size	20
Images for training	9000
Images for testing	901
Number of iterations	200
Activation function	ReLu
Loss function	Cross entropy
Optimization method	Adam
Regularization	None
Learning rate	$1e^{-2}$
**Parameters of CNN**	
Pooling layer	Max pooling
Convolution type	Basic
**Parameters of MLP**	
Layer type	Full connected
Output layer transfer function	pureline

**Table 2:** Number of labels of each class for all maps.

	H	D	UNH	NV	HC	DC	UNHC	HVC	Total
**Bicentenario Park**	2677	7917	376	5270	5	162	247	146	16,800
**ESCOM-IPN**	5011	5241	204	10,829	14	76	134	91	21,600
**Bonito Ecatepec**	321	106	41	5350	2	18	74	88	6000
**Forest area**	2258	1429	259	1008	6	55	242	143	5400

**Table 3:** Number of square meters analyzed by the drone.

	H	D	UNH	NV	HC	DC	UNHC	HVC	Total
**Bicentenario Park**	18,364.22	54,310.60	2579.36	36,152.20	34.30	1111.32	1879.64	1,007.45	115,439.10
**ESCOM-IPN**	34,376.46	35,953.26	1399.44	74,287.94	96.04	522.36	919.24	624.26	148,179.00
**Bonito Ecatepec**	2202.06	727.16	281.26	36,701.00	13.72	123.48	507.64	603.68	41,160.00
**Forested area**	15,489.88	9803.00	1776.74	6914.88	41.16	377.30	1660.12	981.00	37,044.08

**Table 4:** Contamination percentages obtained by our system.

	Biomass	Contamination
**Bicentenario park**	67.76	3.64
**ESCOM-IPN**	47.34	1.64
**Bonito Ecatepec**	9.37	16.73
**Forested area**	78.69	7.13

**Table 5:** Related works using the phrase “terrain classification” as search criteria.

	Image Source:			It Is Classified Based on or with the Objective of:					Uses	Test
Authors	UAV	Terrain (Vehicle)	Satellite	Health	Contamination	Texture	Counting	Disasters	NDVI	Accurancy
Ours	X	-	-	X	X	-	-	-	-	72%
[30]	-	-	X	-	-	X	-	-	-	73.61%
[35]	-	X	-	-	-	X	-	-	-	69.6%
[34]	X	-	-	X	-	-	-	-	-	-
[36]	X	-	-	-	-	-	X	-	-	-
[37]	X	-	-	-	-	-	-	X	X	-
[38]	-	X	-	-	-	X	-	-	-	80%
[39]	-	X	-	-	-	-	-	X	-	-
[40]	-	-	X	-	-	-	-	X	-	-
[41]	-	X	X	X	-	-	-	-	X	-
[42]	-	-	-	-	-	X	-	-	-	95.63%
